# Supplementary figures and images for: Urbanization and health: The effects of the built environment on chronic disease risk factors among women in Tanzania
Source: PLoS One. 2020 Nov 3;15(11):e0241810. doi: 10.1371/journal.pone.0241810 (PMC7608895; doi:10.1371/journal.pone.0241810)

S1 Fig: Degree of urbanicity across wealth categories, by urban/rural classification


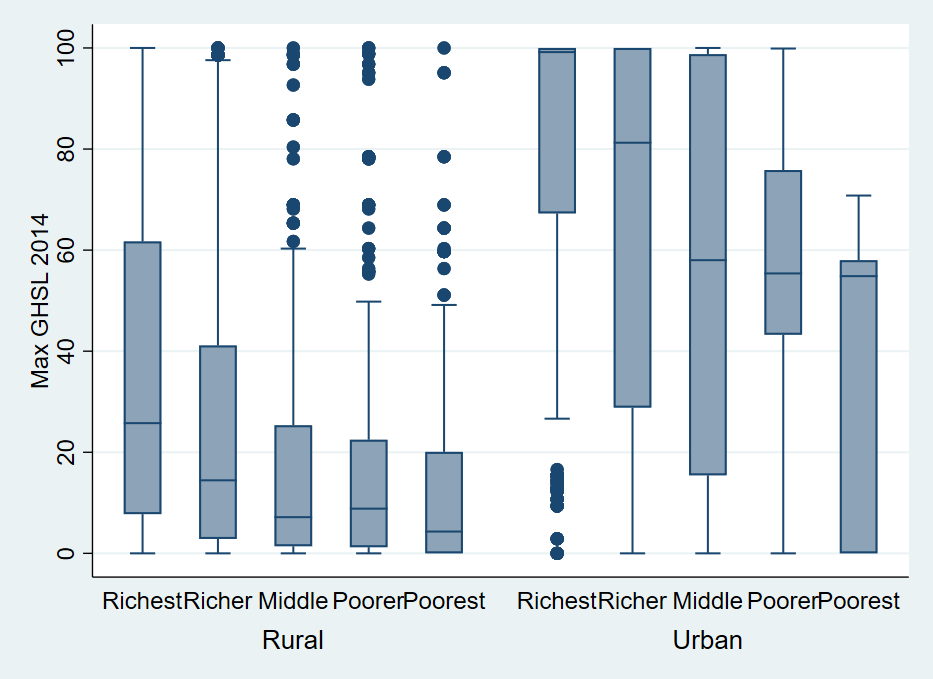

Supplement: S1 Fig — (DOCX) [file pone.0241810.s001.docx]
